# Mechanistic Insights into Axenfeld–Rieger Syndrome from Zebrafish *foxc1* and *pitx2* Mutants

**DOI:** 10.3390/ijms221810001

**Published:** 2021-09-16

**Authors:** Curtis R. French

**Affiliations:** Division of Biomedical Sciences, Faculty of Medicine, Memorial University of Newfoundland and Labrador, St. John’s, NL A1B 3V6, Canada; curtis.french@med.mun.ca; Tel.: +1-709-864-6503

**Keywords:** Axenfeld–Rieger syndrome, *foxc1*, *pitx2*, anterior segment, vasculature, heart

## Abstract

Axenfeld–Rieger syndrome (ARS) encompasses a group of developmental disorders that affect the anterior segment of the eye, as well as systemic developmental defects in some patients. Malformation of the ocular anterior segment often leads to secondary glaucoma, while some patients also present with cardiovascular malformations, craniofacial and dental abnormalities and additional periumbilical skin. Genes that encode two transcription factors, *FOXC1* and *PITX2*, account for almost half of known cases, while the genetic lesions in the remaining cases remain unresolved. Given the genetic similarity between zebrafish and humans, as well as robust antisense inhibition and gene editing technologies available for use in these animals, loss of function zebrafish models for ARS have been created and shed light on the mechanism(s) whereby mutations in these two transcription factors cause such a wide array of developmental phenotypes. This review summarizes the published phenotypes in zebrafish *foxc1* and *pitx2* loss of function models and discusses possible mechanisms that may be used to target pharmaceutical development and therapeutic interventions.

## 1. Axenfeld–Rieger Syndrome

Axenfeld–Rieger syndrome (ARS) is a clinically heterogeneous disorder characterized by ocular anomalies with systemic multi-organ system involvement in some patients. This relatively rare disorder, with a prevalence of 1 in 50,000–100,000 live births [1], presents with structural defects in the ocular anterior segment leading to an early onset glaucoma in about 50% of patients. A subset of patients with ARS have developmental anomalies in other tissues and organs that include craniofacial and dental abnormalities [2,3], cerebral vasculature defects that increase in stroke risk [4], hydrocephalus and Dandy-Walker malformation [5,6], and cardiac developmental defects including aberrant formation of valves and the outflow tract [7,8]. Defects in the pituitary gland with secondary endocrinological conditions can also result [9], as can deficits in the auditory system leading to sensorineural hearing loss [10,11,12] and redundant periumbilical skin is also observed in some cases [13,14]. Clinically, ARS is defined by three subtypes assigned by the presence or absence of systemic anomalies in addition to ocular developmental defects, and correlate with mutations in known ARS causing genes. For an in-depth review of phenotypes associated with ARS, please refer to Seifi et al. [1].

ARS is inherited as an autosomal dominant disorder, with the genetic lesion defined in approximately 40% of cases. Mutations or copy number variation (CNVs) in two genes identified through a variety of family-based studies account for ARS with fully penetrant ocular manifestations; *FORKHEAD BOX C1* (*FOXC1*) and *PAIRED-LIKE HOMOEDOMAIN* (*PITX2*). DNA lesions involving *PITX2* result in ARS type I, in which patients have fully penetrant ocular phenotypes often observed with craniofacial and dental anomalies. Mutations in *FOXC1* result in ARS type III, defined by fully penetrant ocular phenotypes often observed with cardiovascular defects and sensorineural hearing loss. While linkage analysis supports an additional gene causing ARS type II on chromosome 13q14 [15], no causative gene has yet been identified. FOXC1 and PITX2 are transcription factors from the forkhead and homeodomain families, respectively, and can regulate gene transcription independently or physically interact on DNA to co-regulate gene transcription [16]. This complex relationship likely accounts for the phenotypic differences and ARS classifications in patients with mutations in one of the two causative genes. Mutations in the *CYP1B1* gene have been found in a single family with ARS [17], and while a relatively common cause of congenital glaucoma is some populations [18,19], mutations in *CYP1B1* appear to be an extremely rare cause of ARS.

The zebrafish (*Danio rerio*) has provided valuable mechanistic insights into ARS disease etiology. The zebrafish genome contains two homologues of the *FOXC1* gene denoted *foxc1a* and *foxc1b*, arising from an ancient duplication in the teleost lineage, and one homologue of *PITX2* (*pitx2*). Gene expression studies using in situ hybridization have highlighted specific cell types that require the expression of *foxc1* and *pitx2* for normal ocular development. The use of morpholino based antisense inhibition and genome editing have produced zebrafish strains that mimic ARS phenotypes and have provided novel mechanistic data highlighting downstream genes and signaling pathways that are required for ocular and systemic manifestations of the syndrome. This review will focus on the role of *foxc1* and *pitx2* in the regulation of genes and signaling pathways that regulate formation of the eye, cardiovascular system, and craniofacial skeleton, as defined by loss of function zebrafish mutant strains or antisense inhibition data.

## 2. Expression of ARS Genes in Zebrafish

**Figure 1:**
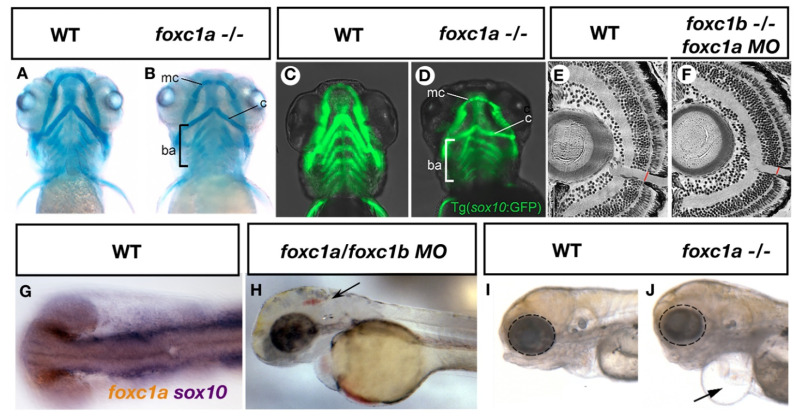
ARS related phenotypes in zebrafish resulting from loss of *foxc1* function. Mutation of *foxc1a* results in hypoplasia of the Meckel’s cartilage (mc), Ceratohyal cartilages (c) and disorganization of the branchial arches (ba) as assessed by alcian blue (cartilage) staining (**A**,**B**) [27]. Such defects may result in part due to decreased *sox10* positive neural crest cells observed in these tissues (**C**,**D**) [27]. Injection of *foxc1a* morpholinos into *foxc1b* homozygous mutants reveals a thinner optic nerve when compared to controls (**E**,**F**) [29]. Expression of *foxc1a* overlaps with *sox10* expression in the periocular mesenchyme (**G**, previously unpublished data from the French lab) while inhibition of both *foxc1a* and *foxc1b* paralogs causes cerebral hemorrhages (arrow, **H**) [4]. Homozygous *foxc1a* mutants display microphthalmia (dotted circle), cardiac edema (arrow) and jaw hypoplasia in lateral brightfield images (**I**,**J**) [27].

At early stages of development, zebrafish express *foxc1a* and *foxc1b* in overlapping domains in neural crest cells [4,20] (Figure 1G, (*foxc1a*)) as well as the lateral plate mesoderm (LPM) [21]. Neural crest cells contribute to the periocular mesenchyme (POM)—a set of cells that are required for closure of the optic fissure and development of the ocular anterior segment [22]. Neural crest cells also populate the central nervous system, craniofacial skeleton, smooth muscle cells of the cerebral vasculature, as well as the cardiac outflow tract and heart valves [23,24,25]. This expression pattern highlights the important role of neural crest cells in development and helps to explain the broad range of phenotypes observed in patients with ARS. The LPM also contributes to the development the heart and cardiovascular system and thus defects in this tissue arising from FOXC1 dysfunction may contribute to cardiovascular anomalies observed in ARS patients [21,26,27]. Although typically known as a developmental disease, *foxc1a* continues to be expressed in the adult zebrafish eye, including the anterior segment and retinal ganglion cell layer [28], possibly contributing to maintenance of adult ocular tissues.

**Figure 2:**
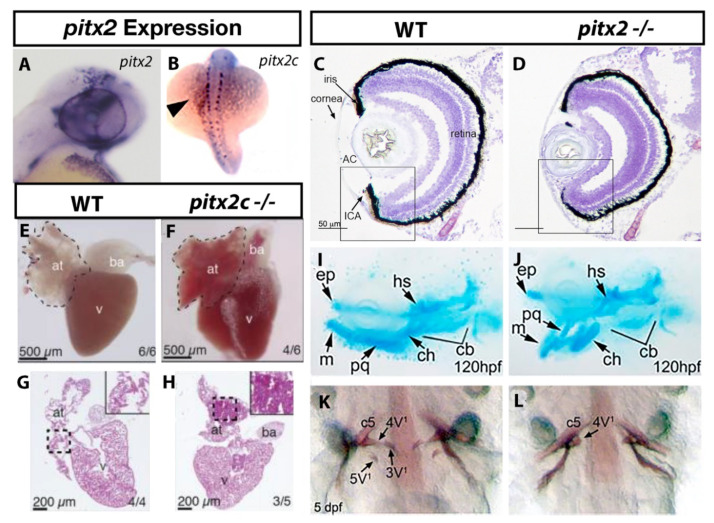
ARS related phenotypes in zebrafish due to loss of *pitx2* function. Expression of *pitx2a* is found within the brain and periocular mesenchyme (**A**) [22] while *pitx2c* is observed in a “left sided” pattern in the lateral plate mesoderm (**B**) [27]. The anterior ocular chamber is reduced in *pitx2* homozygous mutants when compared to wildtype siblings (**C**,**D**) [33]. Homozygous *pitx2c* mutants display hypoplastic cardiac atria and increased fibrosis when compared to wildtype siblings (**E**–**H**) [35]. Underdeveloped jaw cartilages are also observed in *pitx2* mutants (**I**,**J**) [30] as is delayed or absent pharyngeal tooth (3V^1^ and 5V^1^) development (**K**,**L**) [33]. Abbreviations: atrium (at), bulbus arteriosus (ba), ventricle (v), Anterior, segment (AC), ethmoid plate (ep), hyosympletic (hs), Meckel’s cartilage (mc), palatoquadrate (pq), ceratohyal (ch), ceratobranchial (cb).

The zebrafish genome contains a single *pitx2* gene that encodes two isoforms via alternative splicing that correspond to human *PITX2A* and *PITX2C*. The expression of *pitx2a* is found in partially overlapping domains with *foxc1a* and *foxc1b* in developing zebrafish embryos. As early as 24 h post fertilization (hpf), *pitx2* expression is observed in the periocular mesenchyme [22] (Figure 2A), providing an opportunity for co-regulation of gene expression with Foxc1a and Foxc1b. Like *foxc1a* and *foxc1b*, *pitx2* is expressed in the neural crest derived tissues of the pharyngeal arches that contribute to the craniofacial skeleton [30]. Additional expression in the dental epithelium and tooth placodes [31,32] likely accounts for dental phenotypes in ARS type 1 patients. Asymmetric expression of the *pitx2c* isoform in the lateral plate mesoderm [33] (Figure 2B) and expression of *pitx2a* in the neural crest [34], may contribute to cardiac outflow tract defects and valve incompetence observed in a subset of these ARS patients.

## 3. Ocular Related Phenotypes in *foxc1a* and *foxc1b* Mutants

The most distinguishing feature of Axenfeld–Rieger Syndrome is the developmental abnormalities in the ocular anterior segment, leading to an increased risk for early onset glaucoma. Given the ability to create heritable loss of function mutations in zebrafish geusing genome editing techniques, as well as robust antisense (morpholino) inhibition, a number of zebrafish models have been reported that recapitulate the phenotypes of *FOXC1*-attributable ARS patients. Published alleles of zebrafish *foxc1a* and *foxc1b* mutations can be found in Table 1.

The zebrafish anterior segment begins to develop as early as 26 hpf when the lens vesicle detaches from the surface ectoderm [45]. By 3 days post fertilization (dpf), rudimentary structures of the anterior segment are present and will continue to develop through the first month of life. Zebrafish with homozygous mutations in *foxc1a* and *foxc1b* display underdeveloped or absent anterior segments beginning around 3 dpf [39]. Additional ocular phenotypes, including microphthalmia (Figure 1I,J) and coloboma are often observed in double homozygous *foxc1a*/*foxc1b* zebrafish, which are typically not observed in ARS patients. This discrepancy may be the result of complete loss of Foxc1 function in double homozygotes, compared to heterozygous mutations or copy number variations that define ARS. Heterozygous mutations of both *foxc1a* and *foxc1b* in zebrafish do not alter early development of the anterior segment, however, published analyses only describe assays performed up to 6 dpf and thus additional analyses on older heterozygous larvae or adults when the anterior segment has matured, could potentially reveal anterior segment anomalies consistent with ARS. Loss of Foxc1 function in zebrafish also leads to endophenotypes of glaucoma, including reduced number of retinal ganglion cells and thinner optic nerves [29] (Figure 1E,F), providing a valuable model to study the increased glaucoma risk associated with ARS.

Given the similarity of phenotypes in zebrafish *foxc1* mutants and patients with *FOXC1*-attributable ARS, mechanistic insights into disease pathology can be gained. The underdeveloped or absent anterior segment in zebrafish *foxc1* mutants likely results from the role of *foxc1* in regulating the migration and survival of neural crest cells that populate the periocular mesenchyme and subsequently contribute to the iris stroma and cornea [46]. Loss of *foxc1* causes migration defects and increased cell death of *sox10* positive neural crest cells [4] that contribute to these cell populations.

Certainly, the observed anterior segment defects in *foxc1* zebrafish mutants could contribute to mechanism of glaucoma development, given the role of the anterior segment in regulation of intraocular pressure (IOP). However, many ARS patients have normal tension glaucoma (NTG), defined as having normal intraocular pressure, and thus other mechanisms are likely involved. For example, a number of studies have demonstrated that *foxc1* regulates the formation of the zebrafish hyaloid and retinal vasculature [39,47]. Defects in embryonic ocular vasculature could reduce trophic support in the eye or allow the build-up of waste products, but how this may specifically affect the RGCs and the optic nerve remains to be determined. Additionally, the reduced numbers of RGCs observed due to loss of Foxc1 function in zebrafish occurred as the result of reduced expression of *atonal homolog 7* (*atoh7*). Atoh7 is required for RCG differentiation during embryonic development [48,49], and mutations in *ATOH7* in human populations are associated with persistent hyperplasia of the primary vitreous [50] whereby failure of fetal ocular vascular to regress inhibits the development of adult intrinsic retinal vasculature. Thus, the regulation of *atoh7* expression via Foxc1 could account for ocular vascular defects in ARS patients and could influence the development of glaucoma independent of, or in combination with, potential increased IOP caused by the anterior segment dysgenesis. Variants in *ATOH7* have also been associated with primary open angle glaucoma [51] and endophenotypes of glaucoma such as optic disc size [52,53,54], indicating its key role in maintaining these structures. This further supports the role for Foxc1 and its transcriptional regulation of *atoh7* as a key contributor of RGC and optic nerve health.

In addition to *atoh7*, other genetic targets regulated by Foxc1a/Foxc1b in zebrafish have been identified that may shed light on potential mechanisms of glaucoma development. Foxc1a/Foxc1b regulates the expression of *FOXO1A*/*foxo1a*, a gene expressed in the zebrafish POM that mediates the response to oxidative stress [55]. Manipulation of either *foxc1a* or *foxo1a* resulted in aberrant responses to increased oxidative stress and increased cell death in the eye [55], indicating that impaired response to oxidative stress could also be a key facet of glaucoma development due to loss of *FOXC1*. Foxc1a also regulates the expression of *fgf19* [29,56], another gene highly expressed in the zebrafish POM with roles in retinal and lens development [57,58]. Mutations in genes such as *foxo1a* and *fgf19* have not been identified in ARS or glaucoma patients and thus the dysfunction of either gene alone likely cannot cause overt visual defects, however, their reduced expression due to loss of Foxc1 function, as well as other yet to be identified downstream targets, likely contribute to the complex etiology of RGC loss in *FOXC1*-attributable ARS patients.

## 4. Craniofacial Defects in Zebrafish *foxc1a* and *foxc1b* Mutants

Zebrafish *foxc1a* mutants, as well as *foxc1a/foxc1b* double mutants, have craniofacial defects consistent with abnormalities in the facial structure of ARS patients. In these patients, hypertelorism (increased space between the eyes) and a prominent forehead are often described. In zebrafish, *foxc1a* and *foxc1b* are expressed in the neural crest cells that populate the first and second pharyngeal arches that give rise to anterior jaw structures. Mutation of *foxc1a* alone results in craniofacial dysmorphism [27,37] including underdeveloped symplectic cartilage and like many ocular phenotypes observed in these mutants, severity increased in double *foxc1a/foxc1b* mutants. Phenotypes in double homozygous mutants included under-developed palatoquadrate and hyomandibula cartilages [37], demonstrating that *foxc1a* and *foxc1b* play critical roles in the development of anterior facial cartilages (Figure 1A–D, *foxc1a*). Analysis of genetic targets downstream of *foxc1* revealed both *foxc1a* and *foxc1b* regulate Sox9-dependent expression of cartilage specification genes, accounting for reduced jaw structures in these animals. While Foxc1 does not directly regulate *sox9* expression, loss of *foxc1* paralogs in zebrafish causes a decrease in chromatin accessibility for transcription factors such as Sox9 in chondrocytes [38], and thus supports the hypothesis that Foxc1 drives cartilage development through a chromatin remodeling mechanism.

Analysis of heterozygous *foxc1a* mutants in conjunction with homozygous loss of *foxc1b* (which survive to adulthood) demonstrates craniofacial abnormalities in adults that include a misshapen head that closely mimics that of ARS patients, as well as mandibular retrognathia and dorsally positioned eyes [39]. Combined with larval-based studies, these data demonstrate that Foxc1 regulates chromatin accessibility in chondrocytes to shape the jaw and head in zebrafish, with such mechanisms likely contributing the craniofacial dysmorphia observed in ARS patients with mutations or CNVs involving *FOXC1*.

## 5. Cardiovascular Anomalies in Zebrafish *foxc1* Mutants

Cardiac anomalies have been described in ARS patients with *FOXC1* mutations or CNVs that include hypoplastic ventricular outflow tract morphology, dysplastic arcade mitral valve, and atrial septal defect [7,59]. Within the brain, cerebral small vessel disease (CSVD) has been described, which includes increased perivascular spaces, subclinical infarcts and white matter hyperintensities [4]—all of which increase stroke risk [60]. These clinical phenotypes clearly indicate that *FOXC1* plays an important role in early heart and cerebral vascular development, with such phenotypes recapitulated in zebrafish loss of *foxc1* function models.

In zebrafish, mutation of *foxc1a* alone or in combination with *foxc1b* leads to cardiac phenotypes that include hypoplastic myocardium and ventricular outflow tract, as well as defects in cardiac valve formation that are similar to that observed in patients with *FOXC1*-attributible ARS [21,26]. While zebrafish studies generally support a role or *foxc1* in heart development, reports differ in their analysis of heart morphology, assay different time points, and study different combinations of mutations. For example, Yue et al. [21] demonstrate a hypoplastic myocardium, shorter outflow tract, defective primitive valve leaflets, and cardiac edema at during early larval development (4-5 dpf) of *foxc1a*^−/−^ embryos (cardiac edema in *foxc1*^−/−^ displayed in Figure 1J). Ferre-Fernandez et al. [39] assayed cardiac trabecular zone thickness, outflow tract and AV valve morphology in *foxc1a^+/^*^−^; *foxc1b*^−/−^ embryos at 6 dpf and found no difference. Compact zone thickness, however, was significantly larger in these fish when compared to wildtype siblings. While these studies support a role for *foxc1* in heart development, they indicate that homozygous loss of *foxc1a* is required to induce heart defects similar to ARS patients, who typically have heterozygous mutations or CNVs involving *FOXC1*.

Utilizing *foxc1* homozygous loss of function embryos, a role for this gene in regulating cardiac progenitor specification and atrioventricular canal (AVC) formation has been proposed. Such studies show that Foxc1a directly binds to the promoter of *nkx2.5* [26], a gene required for cardiac progenitor specification in the lateral plate mesoderm (LPM). The reduced expression of *nkx2.5* in zebrafish *foxc1* homozygous mutants provides mechanistic insight into the hypoplastic myocardium observed in ARS patients. Furthermore, genes expressed specifically in the AVC are downregulated in *foxc1* homozygous mutants, including *notch1b*, *tbx2b* and *bmp4*, providing additional genetic targets that downstream of *foxc1* that may contribute to cardiac defects. While *foxc1a* is expressed in LPM, the heart also receives a contribution of cells from the neural crest, and thus *foxc1* expression in neural crest cells could also influence the presence and severity of cardiac phenotypes observed in ARS patients and zebrafish *foxc1* mutants.

As CSVD in ARS patients increases stroke risk, analysis of cerebral vasculature due to loss of *foxc1* function in zebrafish has been undertaken. Combined morpholino inhibition of *foxc1a* and *foxc1b* causes cerebral hemorrhages [4] (Figure 1H), a phenotype that is recapitulated in mutant strains [27]. A reduction in neural crest derived pericytes that associate with nascent cerebral vasculature [4,42] is observed, resulting from aberrant neural crest cell migration to the head and increased cell death in *sox10* positive neural crest cell populations. Furthermore, defects in cerebral angiogenesis [39] have been reported, including incomplete connections between the earliest developing cerebral arteries that likely contribute to the hemorrhagic phenotype.

## 6. ARS-Related Defects in Zebrafish *pitx2* Mutants

Like *FOXC1*, mutation of *PITX2* results in ARS with early onset glaucoma in many patients. In zebrafish, both morpholino inhibition or mutation of *pitx2* results in decreased size of the anterior segment during larval development [30,33,43] (Figure 2C,D). Five-day old *pitx2* homozygous mutant larvae have smaller eyes, malformation of the iridocorneal angle, and increased mesenchyme thickness around the cornea with such phenotypes largely phenocopied by morpholino inhibition of *pitx2*. Interestingly, a four-generation pedigree involving anterior segment dysgenesis (ASD) with early onset glaucoma (a diagnosis similar to ARS) identified a 748 kb deletion containing a conserved *PITX2* regulatory element [61]. Removal of this orthologous region using genome editing in zebrafish reduced *pitx2* expression and like mutation of the gene itself, resulted in reduced or shallow anterior chambers. These data clearly demonstrate that mutation of *pitx2*, or genomic modifications that alter *pitx2* expression result in anterior segment defects reminiscent of ARS. Although no direct quantification of optic nerve morphology or RGC number has been undertaken, these data demonstrate that mutant *pitx2* zebrafish represent an ideal model for subsequent studies that focus on the mechanism by which glaucoma may result in patients with *PITX2*-attributable ARS.

Analysis of genetic targets in the eye demonstrates that Pitx2 deficiency reduces the expression of the Wnt ligands (*wnt3*, *wnt4a*, *wnt6b*, *wnt7aa*, *wnt9b* and *wnt10a*) [43] as well as the Wnt antagonist *dkk2* in the anterior segment of the eye [30]. A role for Wnt signaling in eye development has been hypothesized, and thus a regulation of Wnt signaling via Pitx2 could represent a key facet of the ocular phenotypes observed in zebrafish *pitx2* mutants. Wnt signaling plays a key role in the development of neural crest cells in part through regulation of *foxd3* and *sox10* [25,62] that are required for the earliest events of neural crest cell induction. Given *pitx2* expression in early neural crest cells, and its continued expression in the adult anterior segment [28], *pitx2* is likely required for development and maintenance of the anterior segment in part through a Wnt dependent mechanism.

Analysis of additional ocular targets due to *pitx2* depletion demonstrate normal expression of *pax6a* that is required for anterior segment development [30]. While no effect on the expression of the anterior segment marker *pax6a* was observed, it has been shown that *pitx2* expression is regulated by Pax6a/b in zebrafish [28] which may contribute to the anterior segment phenotypes (aniridia, Peter’s anomaly) observed in patients and zebrafish with *PAX6/pax6* mutations [63]. The expression of crystallins required for lens development were also unchanged due to depletion of Pitx2, however, blood vessel defects are highlighted in the embryonic hyaloid vasculature and thus like *foxc1*, *pitx2* may play an important role in early trophic support of the lens and anterior segment structures early in development.

## 7. Craniofacial Defects Due to Loss of *pitx2*

Patients with type I ARS typically have dysmorphic craniofacial features that include maxillary and dental hypoplasia, in addition to the anterior segment ocular phenotypes that define ARS. Similar to *foxc1*, disruption of *pitx2* using antisense morpholinos or with genome editing induced mutations disrupts pharyngeal arch cartilage and jaw formation. Specific defects were observed in the Meckel’s and ceratohyal cartilages, which were under-developed and positioned abnormally [30,43] (Figure 2I,J). Analysis of *sox10* positive neural crest cells that line the pharyngeal arches and jaw elements demonstrate reduced cell number [36], indicating craniofacial dysmorphism in *foxc1* and *pitx2* depleted zebrafish likely involves overlapping mechanisms of decreased neural crest cell migration and survival.

Zebrafish develop pharyngeal teeth that are highly similar to the oral teeth of humans, and mutations that disrupt the DNA binding of Pitx2 in zebrafish cause reduced or absent tooth production [33] (Figure 2K,L)-a common phenotype observed in *PITX2*-attributable ARS. *pitx2* is the earliest expressed transcription factor in tooth bud epithelium and continues to be expressed through tooth development in mice [64] and mammalian cell culture studies demonstrate that Pitx2 acts directly on the promoter of *dlx2* [65,66], a gene required for tooth development. In zebrafish, morpholino inhibition of *pitx2* reduces *dlx2a* expression in the posterior pharyngeal arches [30], demonstrating the conservation of transcription factors that are required for tooth development between fish and mammals and that zebrafish can serve as a useful model to understand defects in tooth development due to loss of *PITX2* in ARS patients.

## 8. Cardiovascular Defects Due to Depletion of *pitx2* in Zebrafish

Like humans, the zebrafish *pitx2* gene encodes multiple isoforms of the Pitx2 protein produced by the use of different promoters and alternative splicing. At least four transcripts encoding four isoforms are present in humans (*PITX2a*, *PITX2b*, *PITX2c*, *PITX2d*) [67] with two homologous isoforms currently annotated in the zebrafish genome (*pitx2a* and *pitx2c*) [33]. During early development, *pitx2a* is predominantly expressed in neural crest cells and thus mutations that affect this isoform may result in ocular, craniofacial, and cardiovascular anomalies associated with ARS, while *pitx2c* is predominately expressed in the lateral plate mesoderm and the heart, which could contribute to cardiac defects observed in some ARS patients. Patients with PITX2-attributable ARS may thus present with or without cardiac defects that may depend in part on the location of the disease-causing mutations [67,68]. Mutant zebrafish have been generated that specifically alter one (*pitx2a*) or both isoforms (*pitx2a* and *pitx2c*) in order to identify similar defects in fish, and to gain mechanistic understanding of such phenotypes in patients and animal models.

Mutations in zebrafish that affect both isoforms (or just *pitx2c*) did not result in cardiac defects consistent with *PITX2*-attributable ARS in one study, although asymmetric looping and overall morphology were the only phenotypes tested [33]. A second study analyzed adult heart morphology in a *pitx2c* specific mutant strain and uncovered cardiomyopathy with fibrosis (Figure 2E–H) and arrythmias, particularly when the fish are stressed [35]. This is consistent with cardiac defects observed in some ARS patients and with the known role of *PITX2* as a causative gene for atrial fibrillation [69,70]. While morphological defects (Figure 2E–H) and arrythmias were analyzed in adult hearts, analysis of gene expression in larval hearts revealed dysregulation of genes involved in mitochondrial function and points toward a mechanism whereby altered cellular metabolism in the heart could contribute to later heart dysfunction. Lastly, while *foxc1* regulates expression of *nxk2.5* in the lateral plate mesoderm, *nkx2.5* is required for maintenance of *pitx2* expression in this area [71], highlighting a potential overlap in mechanism between *foxc1* and *pitx2* in regulating heart development.

## 9. Summary

Analysis of zebrafish *foxc1* and *pitx2* loss of function models provides understanding of the mechanisms that lead to most ARS related phenotypes. Both genes when mutated in zebrafish result in defects consistent with mammalian ARS models and patient phenotypes. Zebrafish ARS mutants have ocular anterior chamber defects, and *foxc1* mutants additionally display endophenotypes of glaucoma. Disruption of *foxc1* or *pitx2* in zebrafish display craniofacial anomalies consistent with ARS, as well as cardiovascular defects that are often observed in patients. *pitx2* mutants additionally display tooth hypoplasia that is often observed in Type 1 ARS. Mechanisms involving downstream gene regulation are beginning to be uncovered using homozygous embryos and larvae, however, analysis of adult phenotypes in heterozygous mutants is somewhat lacking in the literature. The continued analysis of such mutants will reveal novel insights into disease mechanisms, and given the utility of zebrafish for translational research, pharmaceutical approaches using high-throughput drug screening for phenotypic rescue provides a path toward testing therapeutic interventions.

## Figures and Tables

**Table 1:** Published mutant alleles for zebrafish ARS models.

Gene	Allele	Mutation	Published Phenotypes	Reference
*foxc1a*	*nju18*	9 bp del + 2 bp ins	Somite patterning defects	[36]
*foxc1a*	*el542*	5 bp del	Jaw hypoplasia	[37,38]
*foxc1a*	*mw711*	7 bp del	Anterior segment dysgenesis	[39]
			Cardiac defects	
*foxc1a*	*p162*	Nonsense	Fin axon pathfinding defects	[40]
			Vascular defects	[41]
*foxc1a*	*ua1017*	7 bp del	Ocular, vascular, and	[27,29,42]
			L/R patterning defects	
*foxc1b*	*el620*	101 bp deleted	Increases severity of *foxc1a*	[37,38]
			Mutant phenotypes	
*foxc1b*	*mw712*	1 bp inserted	Increases severity of *foxc1a*	[39]
			Mutant phenotypes	
*foxc1b*	*mw713*	1 bp deleted	Increases severity of *foxc1a*	[39]
			Mutant phenotypes	
*foxc1b*	*ua1018*	40 bp deleted	Hydrocephalus, Increases	[27,29]
			Severity of *foxc1a* mutant	
			Phenotypes	
*pitx2*	*mw709*	8 bp deletion	Anterior segment dysgenesis	[43]
*pitx2(c)*	*syn3*	11 bp deleted	None reported	[33]
*pitx2*	*syn6*	1 bp insertion+	Anterior segment dysgenesis	[33]
		5 bp deleted	Tooth, pituitary defects	
*pitx2*	*syn7*	8 bp deletion	Anterior segment dysgenesis	[33]
			Tooth, pituitary defects	
*pitx2*	*syn15*	18 bp insertion+	Anterior segment dysgenesis	[33]
		7 bp deletion	Tooth, pituitary defects	
*pitx2*	*ups6*	2 bp insertion+	Embryonic axis defects	[35,44]
		6 bp deletion	Cardiac arrhythmia	

## Data Availability

Not applicable.
